# Impact of less social connectedness and fear of COVID-19 test on employees task performance: A multi-mediation model

**DOI:** 10.3389/fpubh.2022.951760

**Published:** 2022-09-13

**Authors:** Yueman Zhang

**Affiliations:** School of Chinese Opera, Shandong University of Arts, Jinan, China

**Keywords:** less social connectedness, testing fear, psychological strain, employee health, employee task performance

## Abstract

The core objective of this study is to examine the impact of less social connectedness and testing fear on employee health. This study also investigates the mediating role of psychological strain between the relationship of less social connectedness, testing fear and employee health. Furthermore, this study also assesses the impact of employee health on employee performance. The study's target audience consisted of employees in the electronics industry in China. The convenience sample method was used in this study to collect data from respondents. Data analysis of this study was performed by using the structural equation modeling technique. The statistical software used for data analysis is Smart PLS 3. The results of this study show that less COVID-19 testing fear has a negatively significant impact on employee health, but less social connectedness has not significant direct impact on employee health. Furthermore, psychological strain was discovered to mediate the relationship between less social connectedness and employee health and testing fear and employee health. In addition, this impact of employee health on employee performance was found significant. This study provides theoretical and practical implications. In the context of practical implications, this study provides valuable insights for the organizational management to develop a healthy and positive working environment and adopt healthy behavior among their employees which ultimately foster their job performance.

## Introduction

Pandemic and other stressful life events can have a detrimental impact on an employee's psychological health and task performance. These psychological health disorders include anxiety, stress, cognitive disorientation, social difficulties, and depression (1). Employees who are isolated because of coronavirus disease 19 (COVID-19) feel anxiety, fear, and dissatisfaction. Likewise, uncertainty due to COVID-19 is linked to substantial changes in daily routines. This uncertainty may lead to increased anxiety, despair, and stress (2). Similarly, Vindegaard and Benros (3) published a comprehensive study about the effects of COVID-19 Pandemic on mental health. Giorgi et al. (4) published a narrative review on COVID-19 related health effects in the workplace.

These studies conclusively proved that COVID-19 has resulted in significant symptoms of distress, anxiousness, and disturbance during sleep. Earlier research has looked into the impacts of work stress on various work practices under normal settings (5). The researchers did not look into the effects of stresses on employee task performance (ETP) under unknown situations like the COVID-19 Pandemic (5). Task performance is linked with an employee's work performance in terms of job-related objectives and duties. Task performance represents an employee's capacity and capability to accomplish the tasks. It is regarded as one of the most important indices of organizational performance (6). ETP contributes to the efficiency, productivity, and better environment of working in the organizations (6). Organizations are always trying to survive and grow. To achieve this growth target, they need high-performing employees. The unpredictable external events impair the well-being of employees. Therefore, it becomes challenging for organizations to stay consistent in their operations (7). Unexpected circumstances like COVID-19 can develop discomfort among employees and affect ETP. Pandemic-related dangers at the workplace not only divert employees' attention away from their tasks, but also jeopardize their survival at work by causing health complications (5). In recent decades, scientific studies have focused on the evaluation of ETP. Occupational stress, job satisfaction, disagreement, punctuality, leadership relationships, health, and other aspects have all been studied extensively in task performance studies across the world (8, 9).

According to research, additional stressors occurred amid pandemics. These stresses are linked to the development of several diseases (10). During the recent Pandemic, health issues have grown increasingly evident (11). Furthermore, during the epidemic, employees of different enterprises experienced greater difficulties maintaining themselves healthy (5). It is worth mentioning that the mental health of employees gets improved when they interact with their colleagues (12). Authorities have employed quarantine, lockdowns, and social distancing measures in an attempt to halt the virus's spread. The people were forced to withdraw from their usual routines and acquire physical distance in a relatively short time. The social, economic, and health-related effects of COVID-19 have now become clear (13). The preventive measures have impacted the nature of social connectedness by limiting social relationships with colleagues and friends. Whereas, social connectivity is an important aspect of human existence (14). Its quantity and quality may have a significant impact on the health of employees (15). There is a lack of empirical research on the impacts of social connectedness of employees during COVID-19. There is a need to evaluate the psychological, behavioral and social repercussions of less social connectedness on employees' health and task performance (16, 17). As per some of the previous studies, workplaces play an influential role in improving employees' health (15). According to recent studies, the frequency and persistence of less social connectedness during COVID-19 at workplaces are higher than in domestic lives (14).

Less social connectedness may raise the risk of unfavorable behavioral, social, and health outcomes (13). For several people, physical distance was also associated with discomfort and anxiety (18). Prior literature has indicated that a lack of social connection, persistent stress, and extended emotions of distress may contribute to greater exhaustion and other health issues (18, 19). The distress is a natural response to a lack of social connection. Uncontrolled social isolation causes unpleasant feelings, which may be harmful to employees' mental and physical health (13). Along with the detrimental effects of less social connectedness, fear of testing during COVID-19 was a prevalent stressor which led to disturbed health of employees (20). There have been very limited studies on behaviors associated with testing of COVID-19 (20).

According to literature, several barriers exist in various regions. For example, developing nations have significant challenges in terms of restricted access and counterfeit kits for testing (20). In developed countries, like United States, where medical insurance may not cover the tests, cost may be an issue (20). Particular communities, like immigrants and non-citizens, are disproportionately affected, as they may fear legal and financial consequences if they test positive. Additionally, regardless of COVID-19 exposure, testing may be restricted to particular criteria, such as just if you have symptoms (21). This is common owing to a shortage of supplies or health workers. There may be delivery and samples transportation challenges in rural places (20).

There are challenges associated with poor communication. This is due to lack of public understanding about symptoms. The particular symptoms necessitate testing, and the people are unaware of the procedure (21). There were also structural impediments identified. Delivering testing and transferring samples from far locations were among the problems. In a less developed nation, structural constraints took the shape of insufficient testing centers and the lengthy time it takes to give findings to those who have been tested (22). There are certain emotional and cognitive obstacles in testing COVID-19. These are influenced by personal factors. Such fears include the worry of being in pain while being tested, a lack of understanding about how to get tested, and the fear of contracting a disease at the testing site (22). The COVID-19 affected the employees of various organizations worldwide. The health, lifestyle, financial, and societal changes in addition to increased morbidity and death were the outcomes of this pandemic (23). It is generally agreed that the psychological health of employees has been impacted due to this. A disturbance of work and lost wages due to lockdowns and limitations have been one of the most prominent indicators of psychological health concerns. Employees experience substantial stress, burnout, anxiety and depression during COVID-19 (23). Employees in many industries also experienced an increase in psychological strain (23). The psychological strains negatively influence the health of employees at any workplace. These strains are generally associated with stresses like less social connectedness and COVID-19 testing fear in this study.

Previously in context of COVID-19, psychological strain proved to be a significant mediator between some socio-ecological elements and quality of life (24). This study left a gap in evaluating the mediating role of psychological strain between stressors and employees' health. To fill this gap, current study prospects the role of psychological strain of employees as a mediator between less social connectedness, COVID-19 testing fear and employees' health. No prior study looked into the impact of COVID-19 testing fear and less social connectedness on employees' health. This posed a huge gap for researchers. Furthermore, in context to less social connectedness and COVID-19 testing fear, impact of employees' health on their task performance was also not studied before. This study tries to fill these gaps by evaluating the impacts of both stresses on employees' health leading to ETP.

The current study tries to find the answers to following questions.

**RQ**_**1**_. What is the possible relationship between a preventive measure such as less social connectedness on employees' health?

**RQ**_**2**_. What is the role of fear associated with COVID-19 testing on employees' health?

**RQ**_**3**_. How does employees' health influence the employees' task performance?

**RQ**_**4**_. How can employees' health be affected due to psychological strain?

## Theoretical support and hypothesis development

This research gets support from Person-Environment fit theory (25). This theory is suitable for examining the perceived discrepancies. According to this theory, congruence, match, or likeness between personal and environmental elements is defined as fit (26). According to studies that examine the relationship between social connectedness and employee health, the match between an employee's desire for social connections and the environment's supplies has a favorable impact on employee health. Employee health improves when the supply of social connection grows in terms of quantity and quality to meet the demands of the employee. Employees feel stress and loneliness when the workplace resources fall short of their demands, resulting in health problems (25). On the other hand, a lot of social connectedness may obstruct the desire for privacy or prevent task performance which needs isolation (25). This kind of excessive social connectedness may harm the employees' health. Support from friends and family has a comparable impact. A higher imbalance between required and given social support is linked to more depression symptoms. Although, the relationship is asymmetrical having depressive symptoms at peak when requirements outweigh supplies (27). Based on the Person-Environment Fit theory, this study looks into reduced social connectedness at work and the health of employees during the COVID-19 epidemic.

This study is related to psychological aspects of the employees and their performance. Previously, studies like this got support from several theories including demands-control model, job demand-resources theory (JDR), conservation of resources theory (COR) and person-environment fit theory. All these theories are related to work stress and have shown impact on work and health related psychology of people (28). The JDR theory looks at how working environments affect workers and how people affect employment conditions. At organizational, team, and individual levels, variables of employees' health and organizational behavior impact each other throughout time (28). The JDR hypothesis describes how job demands can affect an employee's health, behavior, and task performance. Workplace stresses are detected quite precisely based on the emotions and opinions of employees. In a same way, Pandemic related difficulties might be described as disruptions in task performance of employees. The authors look at the COR theory's theoretical underpinnings as well as previous studies that used it to look at anxiety and responsibility in companies and mental health care settings (29). The COR theory is a suggested descriptive framework for exploring how employees get influenced by extreme situations, recognizing such events, and theorizing on how employees collaborate to deal with problems. Burnout, heavy workload, and lack of administrative and institutional resources have all been linked to the use of COR theory (28). In current research, the COVID-19 related stressors include less social connectedness and fear of testing COVID-19 positive. This theory helps in providing theoretical support for these stressors to evaluate their impacts on employees' health and employees task performance.

### Less social connectedness and employees' health

The subjective experience of having strong links to the social sphere is referred to as social connectedness (30). This is based on Lee and Robbins' (30), fundamental concept of a sense of affiliation and interpersonal interactions in their study report. When working in remote places, social connectivity is described as closeness and a feeling of connection with relatives, family, and society in the home environment. Connectedness, according to Hong et al. (31), It is a multifaceted construct that has a key role in enhancing self-esteem, contentment, and optimism. Employee happiness and productivity are affected by social life, particularly for employees working in remote locations (32). Moreover, in distant places, there are fewer institutions and educational places for employees' children to seek a regular education. This also has an impact on mental health and job performance of employees (33). This happens because social connectedness is a basic human need. Employee motivation and health are both influenced by social connectivity. Disruptive interactions with individuals like family members may undoubtedly have serious consequences for overall health (34). Frequent interaction with coworkers and acquaintances is strongly linked to feelings of fulfillment and contentment. The work–family integration study backs up this theory, suggesting that strong family ties can improve emotional responses at work (35). Moreover, researchers noted that working in remote places has a detrimental influence on employee health and productivity (35).

There has been a lot of research going on to reduce the bad effects of less social connectedness on employee's subjective well-being. One of the coping strategies has been proposed as long yearly leave for employees to give them time to connect with their families and friends (36). This strategy may lead to fighting the psychological health degradation of employees. The employees have limited opportunities to connect with their families and friends as they are working in remote areas. There is a need to find ways which may help the employees to work better with sound psychological health. Furthermore, human resource professionals are researching on how to provide flexible timing to employees working in isolated workplace settings (36). All efforts are oriented toward solving this difficulty that might negatively affect employee health leading to poor task performance.

Authorities and researchers have expressed concern that individuals would be socially isolated for lengthy periods of time as a result of worldwide policies. This type of exclusion is characterized as the lack of social relationships, or as a lack of social connectedness (37). There is scarce literature available on the relationship between less social connectedness and mortality. An investigation reveals that the real and desired connectedness has a greater impact on employees' health than the lesser or no social connectedness. When looking at the effects of the COVID-19 outbreak in the workplace, this disparity should be taken into account as a possible avenue (38). The COVID-19 and its preventive measures like social distancing may harm employees' health (38). Following this perspective, the following hypothesis was developed.

***H***_**1**_**.** *Less social connectedness of employees has a significant but negative association with employees' health*

### COVID-19 testing fear and employees' health

Fear is a natural human emotion which intimates about harm (39). The unpleasant sensation has negative consequences for people's overall health. Employees' health is harmed by fear, which leads to an increase in depressive symptoms. This is a mental illness which exacerbates feelings of low morale, sadness, grief, and stress. It negatively impacts an individual's mental health (40). Pandemics instill in the community a feeling of despair throughout time. Nowadays, the corona virus has increased the reactivity of depression amongst employees by accelerating their fear. Employees' depression symptoms were dramatically increased when COVID-19 levels rose, negatively impacting their psychological health (41).

In support of this, a study looking at the psychological symptoms linked to fear of testing COVID-19 positive, found a significant frequency of anxiety and depression among Chinese healthcare employees (42). Employee work performance was significantly impacted by the fear of testing positive for COVID. According to the findings, increasing COVID-19 anxiety caused the health workers to demonstrate poor task performance, dramatically affecting working practices. Fear of testing COVID-19 positive for infectiousness caused frontline employees to take on too much work, which hampered their performance (43). Due to the higher psychological concerns, employees' performance suffered as a result of the increased Fear of COVID-19. Employees' work performance is influenced by their mental health in particular. Employees of many organizations have been forced to execute their responsibilities at all hours of the day and night due to the present epidemic. Undoubtedly, the epidemic increased psychological concerns, making it harder for staff to do their jobs (44). Fear necessitates a protective reaction. Whenever fear becomes unmanageable, it transforms into anxiousness. Increasing COVID-19 positive fear testing has taken a heavy emotional strain on employees' psychological health in recent years, effectively forcing them to work with a panic illness. The COVID-19 dread produced serious health problems, which had an impact on the caregivers' life. Mertens' (45) research backs this and reveals that the Pandemic generated anxiety and panic among the workforces.

Moreover, the spread of corona virus showed the pandemic as a major factor in people's stress symptoms. Stress is a coping mechanism that necessitates physiological, psychological, and cognitive adaptation. Stress affects everyone differently depending on behavioral, physiological, and emotional factors (46). The worry of testing positive for COVID-19 has a major impact on people's mental health, inflicting severe anguish on them. Individuals were negatively affected by increased emotional tiredness, energy loss, and fatigue. It made it harder for them to manage with the COVID-19 pressures (47). Employees reacted negatively to challenging events as a result of the COVID-19 scenario, negatively affecting their psychological well-being. In China, more than 100 million people reported experiencing signs of high stress, according to the survey (47). All these arguments suggest that fear of testing COVID-19 positive may lead to disturbed health of employees so, the authors proposed the following hypothesis.

***H***_**2**_**.** *COVID-19 testing fear has a negative relationship with employees' health*

### Employees' health and employees' task performance

Employee task performance may be described as an employee's effort to complete certain job obligations. According to researchers, employees' task performance is associated with cognitive health of employees (48). The conservation of resources theory (COR) is based on the idea that people want to get, keep, nurture, and defend the assets they care about most. As a result, their health is heavily reliant on the inflow and preservation of essential resources like spouse assistance and work performance. Employees participate in practices to prevent the damaging effect of loss of resources on their health. For years, scholars have tried to figure out how distinct work-family investment needed strategies which affect job performance and happiness (48).

According to some researchers, positive job experiences are related to better emotional health. Employees with greater levels of mental health are able to perform better at work (49). Employee health appears to play a larger influence in generating instead of forecasting variation in task performance. Employees who are in better health have more emotional stability. They are more positive, adaptable, and capable of dealing with problems. Employees' health is directly linked to a variety of beneficial elements of their domestic lives and professional careers (49). This notion must be handled comprehensively rather than temporally to be completely understood. Although it is not a wholly context-dependent phenomena, it may be impacted by ecological, organizational, and social activities and therefore should be addressed. Employee health has been shown to affect employee task performance in previous research. Employee efficiency and performance are linked to their well-being. Employees who are happier make better decisions, have more social behaviors, and have higher performance appraisal (50). Employees must be mentally fit in order to reach desired aims and create predicted outputs, suggesting that their mental attention should be completely on job duties. So, based on this notion that healthy employees may perform better and accomplish their tasks diligently, authors tried to figure out the relationship between employee health and their task performance during COVID-19 pandemic. It is therefore assumed that stressors have negative impact on employee's health while employees' health is directly associated to employee task performance. So, the following hypothesis was built.

***H***_**3**_**.** *Employee health has a strong association with employee's task performance*.

### Psychological strains

Individuals' symptoms of depression were amplified during the Pandemic as psychological strain escalated. These growing public health concerns exacerbated the link between both the COVID-19 spread and anxiety. A rise in psychological strains exacerbated the connection between employee engagement and anxiety. An unfavorable encounter with workers' health led health workers to be concerned about their own mental health (51). As per the findings, patients having severe anxiety experience tiredness, lethargy, and a lack of vitality. Workplace detachment is commonly reported as a result of psychological strain, causing unhappiness among employees. The current Pandemic puts employees of numerous organizations under a great deal of psychological strain (52). Therefore, psychological strains must be examined to match up the demanding expectations of the professions.

Workers were scared by the thought of infecting their relatives and friends during COVID-19. Workplace wellness is critical to providing safe services to customers. Scholars have given positive psychological well-being a lot of thought. They understand the importance of employee health. Employee satisfaction may be influenced by psychological well-being. As a result, data of psychological stress were collected during the COVID-19 phase (53). The study illustrated how unpredictable condition of the outbreak caused workers to give up control over their job. Employees were concerned about the virus's growing infectivity due to its vast dissemination.COVID-19 caused substantial psychological strains that harmed people's health (54). Considering the explanation, the authors conclude that employees had stress-related symptoms during the COVID-19.

Depression, a negative state of mind, has far-reaching implications that affect an employee's motivation. During the outbreak of COVID-19, depression was often noticed among employees of numerous enterprises. COVID-19's elevated complaints rendered the business sector the most sensitive to serious depression. Employee productivity and performance are severely hampered by depression (55). Employees are fatigued and disengaged from their jobs as their depression levels rise. As a result, it may have an influence on their capacity to give high-quality solutions. The goal of a corporate employee is to deliver high-quality service to customers. Perhaps, in order to achieve professional success, workers' psychological well-being must be safeguarded (56). Studies have discovered anxiety as the primary cause of compromised job performance. Anxiety is a distressing emotion which impairs one's mental abilities. Anxiety may lead employees to be concerned about their jobs, thereby it may increase their productivity. While employees' productivity is affected by anxiety, and it hinders their work performance. According to the research, there is a substantial negative association between anxiety and performance of employees (57). All this supported literature suggested that stressors like less social connectedness may produce psychological strain on employees. Similarly, testing fear for COVID-19 positive may also contribute to psychological strains of employees. The result of supported literature also suggest that psychological strain leads to poor health of employees. Employees' health is disturbed due to depression, stress and anxiety which are the contributors of psychological strain. Employees having sound health in return, may help in improving ETP. Therefore, authors suggested the following hypothesis.

**H**_**4**_. *There is a significant but negative mediating association between less social connectedness and employees' health*

**H**_**5.**_ *There is a significant but negative mediating association between fer of testing COVID-19 positive and employees' health*

**Figure 1:**
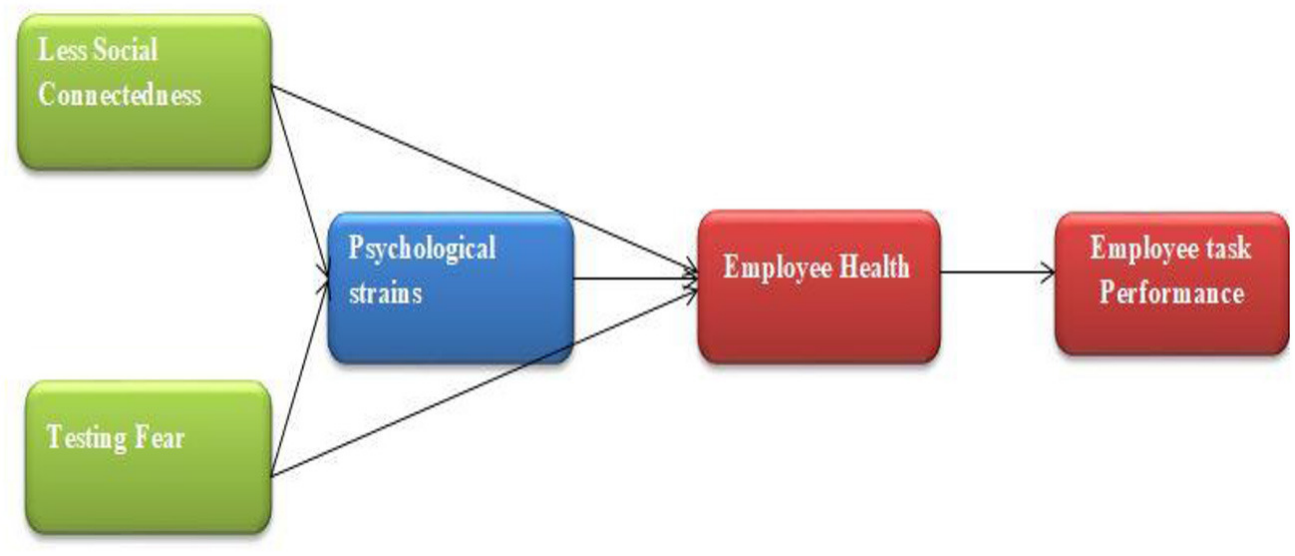
Conceptual framework.

The current study is summarized in the following conceptual framework (see Figure 1).

## Methodology

This study gathered data from employees of various electronics industries in China under a convenient sampling technique for empirical analyses. For data collection, the author first contacted the managers of the electronics industries and had a detailed conversation regarding the purposes of being contacted. After knowing the academic purpose, managers agreed to a face-to-face meeting. The meeting has been fixed as per time and visited personally to meet them. The author explained the study objective and outcomes' usefulness for the organizations in the meeting. The author also promised that the practical implication of this study would be shared with them at their request. In this way, managers showed their consent and agreed and permitted the author for data collection.

The author adopted a questionnaire survey method for data collection. A dual-language questionnaires for data collection has been developed. So for this purpose, the author translated questionnaires into Chinese as well for easy understanding of employees. For translation, the help has been taken from senior researchers, and under their guidance, the questionnaires were translated into the Chinese language for a better understanding of the employees. The author collected sample base data from students for further clarity of the Chinese language. Hence, the to revised the difficulties in this way, and the final version of the questionnaires was ready for distribution. The questionnaires were also developed, including a cover letter. This cover letter trusted the employees that their data would be used only for academic purposes. The cover letter also ensured the employees' about their data confidentiality as individual-level responses would be destroyed, and aggregated results would be revealed. Moreover, the Cover letter also explained that no answers are right and wrong as your true answer would be treated right for this study. Hence, this step boosts the employees' confidence, and they would have filled questionnaires of their own will.

The author also decided to collect data at different waves to avoid common method bias. Hence, to utilized a time lag data approach to questionnaire distribution among employees. For this purpose, the questionnaires were also developed based on a hidden code to recognize the same respondents in all waves. The questionnaires has been distributed in three waves. In the first wave, the author distributed questionnaires regarding independent variables such as less social connectedness and testing fear. In the second wave, the author collected data on mediator variables such as psychological strain and employee health and in the last/third wave, the data has been collected on dependent variables such as employee task performance. In the first wave, the total of 750 questionnaires distributed among employees. Out of 750, the author collected complete and valid 630 questionnaires. In the second wave, the author collected 505 valid and complete questionnaires, and in the last/third wave, the author collected 467 complete questionnaires. Hence, this study is based on a 467 sample size.

### Scales

In this study, respondents' responses were captured using a 5-point Likert scale ranging from 1 (strongly disagree) to 5 (strongly agree). The previously validated items were considered to measure the present study model variables.

#### Less social connectedness

The less social connectedness variable was measured with eight items scale developed by (58) and validated by (59). The sample item included “I feel disconnected from the world around me.” The Cronbach alpha value of construct less social connectedness is 0.906 showing the acceptability of scale.

#### Testing fear

The variable “testing fear” was measured on seven items scale developed by (60) and validated by (61). The sample item included “It makes me uncomfortable to think about coronavirus-19.” The Cronbach alpha value of testing fear is 0.883 showing the acceptability of scale.

#### Psychological strain

The psychological strain variable was measured with eight items scale developed by (62) and validated by (63). The sample item included, “Even at home I often think of my problems at work.” The Cronbach alpha value of psychological strain is 0.885 showing the acceptability of scale.

#### Employee health

The employee health variable was measured with five items scale developed by (64) and validated by (65). The sample item included, “Most of the time, I think my health is not good.” The Cronbach alpha value of employee health is 0.911 showing the acceptability of scale.

#### Employee task performance

This study measured task performance with seven items scale developed by (66) and validated by (67, 68). The sample item included, “I fulfill responsibilities specified in the job description.” The Cronbach alpha value of employee task performance is 0.886 showing the acceptability of scale.

## Results

### Common method bias

The present study incorporated different methods to ensure avoidance of common method bias issues (69). First, for this purpose, this study collected data in three waves as common method bias mostly occurred in cross-sectional studies during single source data collection. The detail of this method application is addressed in the data collection part. Second, this study applied Harman's (70) single factor test to ensure further clarity about common method bias. For this purpose, SPSS software was used, and under this examination, all factor items were forced into one single factor to evaluate variance. As per the output, single factor variance explained < 50% (40.144%), confirming that common method variance is not an issue in this study. Third, this study applied Bagozzi's method (71). According to Bagozzi's, focal study constructs correlations >0.9 shows the presence of common method variance. However, Appendix 1 shows the highest correlation between the two constructs is 0.737. Fourth, collinearity examination was conducted through variance inflation factor (VIF). The outcomes revealed that the highest VIF value is less than 3.3, indicating that model is without a common method bias issue (72).

### Assessment of measurement and structural model

This study utilized the partial least square structural equation modeling (PLS-SEM) method to assess empirical outcomes. The PLS-SEM technique is different from the covariance-based technique (73). PLS-SEM is widely acknowledged because it supports both studies, such as confirmatory and exploratory (74–76). Structural equation modeling (SEM) consists of two methods, i.e., partial least square structural equation modeling (PLS-SEM) and covariance-based structural equation modeling (CB-SEM). PLS-SEM is acknowledged to advance and extend the theory. In contrast, CB-SEM is known for accepting and rejecting the theory (77). Hence, this study assessed model results using Smart PLS software under the PLS-SEM method. PLS-SEM measured data in two parts. The first part considers the measurement model, and the second examines the structural path.

Outcomes related to measurement consist of two different parts such as model reliability and validity. This study assessed the reliability of the present study model through Cronbach alpha, roh-A, composite reliability, and average variance extract (AVE) (77, 78). Table 1 presents the reliability values of model variables. As per the threshold of Cronbach alpha, a value >0.7 is considered appropriate for Cronbach alpha reliability (74). This study models variables (less connectedness, testing fear, psychological strain, employee health, and task performance). Cronbach alpha values are (0.906, 0.883, 0.885, 0.911, and 0.886) according to the given standard as above 0.7, respectively. Hence, Cronbach alpha reliability is achieved in this study. Similarly, the composite reliability should also be >0.7 (79). All variables' composite reliability values are as per the criteria as >0.7. Hence, composite reliability is achieved. The roh-A values are also as per the standard. Thus, all values are accepted (79). Moreover, as per the threshold, AVE values should be above 0.5. Our model variables' AVE values are >0.5. Thus, AVE values are accepted (79).

**Table 1:** Reliability and convergent validity of the study constructs.

**Construct**	**Item**	**Outer loadings**	**VIF**	**Alpha**	**roh-A**	**Composite reliability**	**AVE**
EH	EH1	0.833	2.346	0.911	0.913	0.934	0.739
	EH2	0.876	2.870				
	EH3	0.882	2.916				
	EH4	0.860	2.555				
	EH5	0.846	2.367				
LSC	LSC1	0.763	2.064	0.906	0.907	0.924	0.603
	LSC2	0.754	1.934				
	LSC3	0.746	2.023				
	LSC4	0.812	2.428				
	LSC5	0.789	2.253				
	LSC6	0.811	2.478				
	LSC7	0.776	2.111				
	LSC8	0.759	2.020				
PS	PS1	0.806	2.188	0.885	0.888	0.910	0.593
	PS3	0.731	1.789				
	PS4	0.759	1.897				
	PS5	0.763	1.948				
	PS6	0.783	1.984				
	PS7	0.775	2.078				
	PS8	0.768	1.931				
TF	TF1	0.794	2.085	0.883	0.890	0.912	0.633
	TF2	0.741	1.814				
	TF3	0.792	2.119				
	TF4	0.870	2.907				
	TF5	0.811	2.509				
	TF6	0.759	1.961				
TP	TP1	0.803	2.639	0.886	0.890	0.912	0.635
	TP2	0.780	3.208				
	TP3	0.779	3.245				
	TP4	0.784	2.205				
	TP5	0.826	2.949				
	TP6	0.807	2.489				

EH, Employee Health; LSC, Less Social Connectedness; PS, Psychological Strain; TF, Testing Fear; TP, Task Performance.

**Figure 2:**
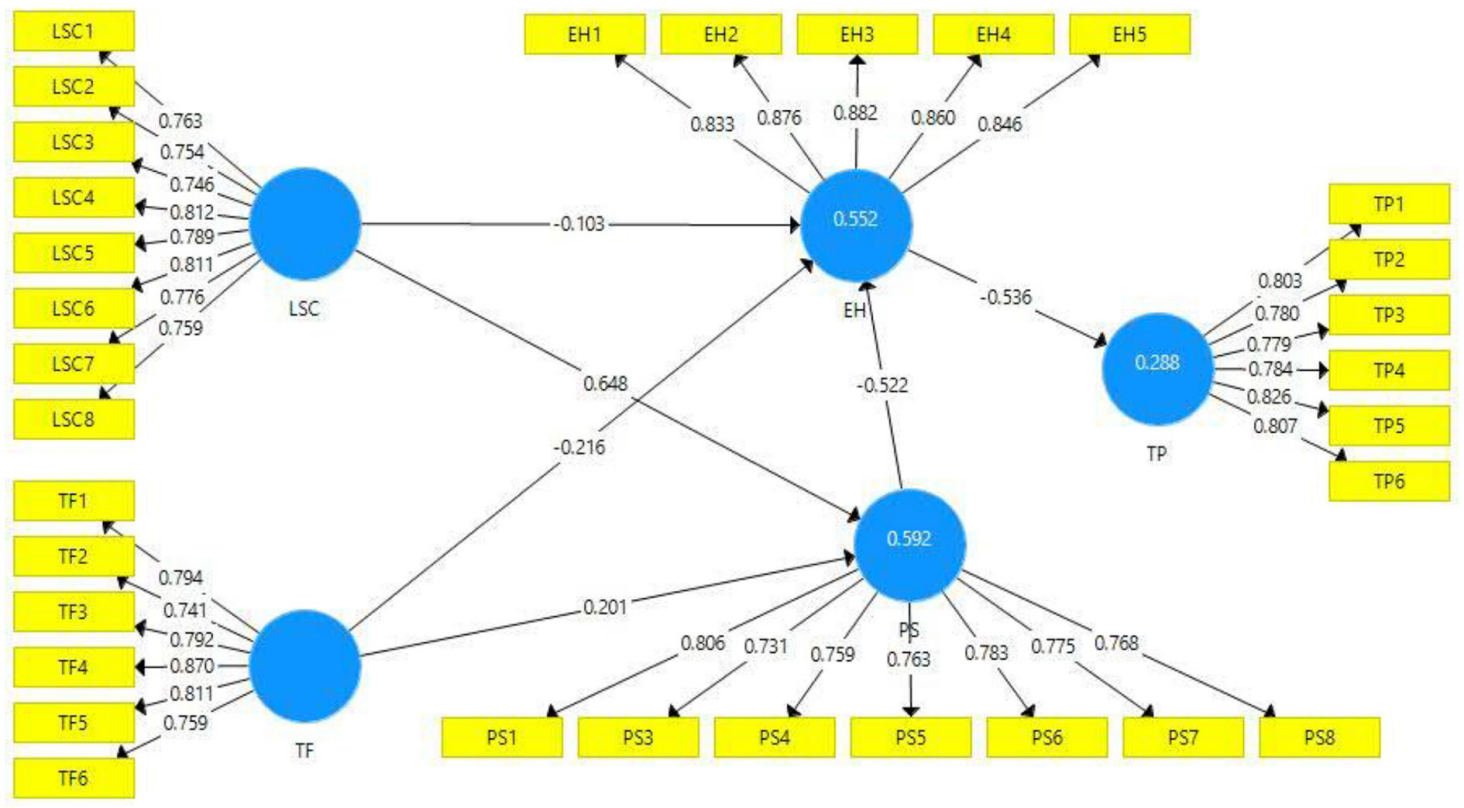
Path estimates.

Outer loading of all the construct items is also depicted in Table 1. The outer loading value is accepted if above 0.7 (68). In the present study model, all variable items have >0.7 outer loadings values (Figure 2) except for psychological strain item PS2, testing fear item TF7, and task performance item TP7, thus deleted. In this way, the model reliability was increased. The variance inflation factor (VIF) values of all variable items are also presented in Table 1. VIF is the source to assess the collinearity issue in the model. A value <0.5 is considered appropriate for the model as it shows the model is without a collinearity issue (79)s. The variable task performance item TP3 has the highest VIF value (3.245). Hence, it confirmed that the present study model is free from collinearity issues.

The model latent construct *R*^2^ examines the model strength. For instance, the value near 0.5 explains moderate strength and above 0.5 shows substantial strength (80). The present study's latent variables psychological strain, employee health, and task performance *R*^2^ values are 0.592, 0.552, and 0.288, respectively, revealing that model has moderate and substantial strength. The model latent constructs Q^2^ values are accepted if greater than zero. The present study's latent constructs have more than zero value. Thus, it is a sign of a significant model.

This study considered widely familiar and accepted methods to measure the model's discriminatory validity. For instance, Fornell-Larcker and hetrotrait-monotriat (HTMT) criteria were applied to evaluate the discriminant validity of the present study model (77). The Fornell-Larcker criterion was measured by taking model variables AVE values square roots (78, 81). Table 2 explains the Fornell-Larcker values of the present study model. As per the standard, the above values in the table column should be greater than the below values (73). The outcomes revealed that the above values in the Table 2 column shown in bold were greater than their below values. Hence Fornell-Larker discriminant validity is achieved. According to the HTMT criterion, values <0.85 is considered appropriate for the model (79). Table 3 explains that the present study model constructs HTMT values according to the given criterion, such as <0.85. Hence HTMT discriminant validity is achieved.

**Table 2:** Discriminant validity (Fornell-Larker-1981 Criteria).

**Construct**	**Employee health**	**Less social connectedness**	**Psychological strain**	**Testing fear**	**Task performance**
Employee health	0.859				
Less social connectedness	−0.604	0.777			
Psychological strain	−0.714	0.750	0.770		
Testing fear	−0.544	0.506	0.529	0.796	
Task performance	−0.536	0.577	0.720	0.375	0.797

**Table 3:** Discriminant validity (HTMT).

**Construct**	**Employee health**	**Less social connectedness**	**Psychological strain**	**Testing fear**	**Task performance**
Employee health	–	–	–	–	–
Less social connectedness	0.660	–	–	–	–
Psychological strain	0.790	0.832	–	–	–
Testing fear	0.601	0.563	0.592	–	–
Task performance	0.585	0.631	0.799	0.415	–

### Hypotheses testing

The present study applied 5,000 samples of the bootstrapping method for the empirical analysis of the model. The direct, indirect, and total path outcomes are presented in Table 4 (77, 78). The hypotheses of the present study were accepted and rejected based on t and *p*-values (78). Table 5 depicts the hypotheses results. According to the proposition of H1 of this study, it assumed that less social connectedness has a negative impact on employee health. The outcomes (*t* = 2.297, *p* ≤ 0.05) revealed that less social connectedness negatively influences employee health. Hence, H1 is accepted. The path value of H1 explains that one unit change in less social connectedness would result in −0.103 change in employee health. Proposition H2 of this study predicted that testing fear has a negative impact on employee health. According to the statistics results (*t* = 5.959, *p* ≤ 0.001), it is confirmed that testing fear negatively influences employee health. For instance, as per path value, one unit change in testing fear would cause a −0.216 change in employee health. Hence, H2 is accepted. H3 of this study proposed a strong association between employee health and employee task performance. The outcomes (*t* = 8.730, *p* ≤ 0.001) confirmed that employee health negatively influences the employee task performance. Such as path value confirmed that one unit change in employee health will cause −0.536 in employee task performance.

**Table 4:** Direct, indirect and total path estimates.

	**Beta**	**SD**	***t***	**Confidence interval (95%)**	***p***
**Direct path**					
EH -> TP	−0.536	0.061	8.730	−0.650 to −0.412	0.000
LSC -> EH	−0.103	0.045	2.297	−0.193 to −0.016	0.022
LSC -> PS	0.648	0.047	13.849	0.546 to 0.730	0.000
PS -> EH	−0.522	0.049	10.614	−0.615 to −0.423	0.000
TF -> EH	−0.216	0.036	5.959	−0.290 to −0.146	0.000
TF -> PS	0.201	0.042	4.805	0.115 to 0.281	0.000
**Indirect path**					
LSC -> PS -> EH	−0.338	0.042	8.040	−0.336 to −0.419	0.000
TF -> PS -> EH	−0.105	0.024	4.344	−0.104 to −0.153	0.000
LSC -> EH -> TP	0.055	0.026	2.162	0.056 to 0.008	0.031
LSC -> PS -> EH -> TP	0.181	0.040	4.590	0.182 to 0.110	0.000
PS -> EH -> TP	0.280	0.051	5.516	0.281 to 0.186	0.000
TF -> PS -> EH -> TP	0.056	0.016	3.517	0.056 to 0.028	0.000
TF -> EH -> TP	0.116	0.022	5.308	0.116 to 0.076	0.000
**Total path**					
EH -> TP	−0.536	0.061	8.730	−0.650 to −0.412	0.000
LSC -> EH	−0.441	0.050	8.818	−0.539 to −0.338	0.000
LSC -> PS	0.648	0.047	13.849	0.546 to 0.730	0.000
LSC -> TP	0.237	0.048	4.878	0.148 to 0.338	0.000
PS -> EH	−0.522	0.049	10.614	−0.615 to −0.423	0.000
PS -> TP	0.280	0.051	5.516	0.186 to 0.383	0.000
TF -> EH	−0.321	0.042	7.592	−0.406 to −0.239	0.000
TF -> PS	0.201	0.042	4.805	0.115 to 0.281	0.000

EH, Employee Health; LSC, Less Social Connectedness; PS, Psychological Strain; TF, Testing Fear; TP, Task Performance.

**Table 5:** Hypotheses testing.

		**Coefficient (Beta)**	**S.D**	***t***	***p***	**Status**
	**Hypotheses**					
H1	Less social connectedness -> Employee health	−0.103	0.045	2.297	0.022	Supported
H2	Testing fear -> Employee health	−0.216	0.036	5.959	0.000	Supported
H3	Employee health -> Task performance	−0.536	0.061	8.730	0.000	Supported
	**Mediation hypotheses**					
H4	Less social connectedness -> Psychological strain -> Employee health	−0.338	0.042	8.040	0.000	Supported
H5	Testing fear -> Psychological strain -> Employee health	−0.105	0.024	4.344	0.000	Supported

The present study also assessed the mediating role of psychological strain as a mediator between less social connectedness and employee health and testing fear and employee health. For this objective, this study proposed H4, which predicts the mediation role of psychological strain between less social connectedness and employee health. According to the statistics outcomes (*t* = 8.040, *p* ≤ 0.001). it is confirmed that psychological strain mediates the relationship between less social connectedness and employee health. The path value (−0.338) confirmed that psychological strain negatively mediates this relationship. Hence, H4 is accepted. The H5 of the present study predicts the mediation role of psychological strain between fear testing and employee health. The outcomes (*t* = 4.344, *p* ≤ 0.001) confirmed that psychological strain mediates the relationship between testing fear and employee health. The path value (−0.105) confirmed that psychological strain negatively mediates this relationship. Hence, H5 is accepted.

## Discussion

This research focused on some of the stressors during the COVID-19 Pandemic. This study aimed to evaluate the impact of stressors on employees' health because it is directly associated with employees' task performance. Employees' task performance is a phenomenon that deals with the productivity and efficiency of employees regarding specific tasks in an organization. This study dealt with employees' responses from the electronic industries of China, which is a progressive sector in the country. The negative consequences of the Pandemic have adversely hit the employees of this sector during the last 2 years (82). According to this research, employees associated with the electronic industry of China are more at risk of contracting health-related concerns as government is unable to implement proper health regulations in this industry. During COVID-19, this industry was also affected like other industries (42).

This study is based on direct relationships of stressors with employees' health. First of all, current research looked into the direct impact of less social connectedness on employees' health. The results showed that less social connectedness reduced employee health (Table 5). Due to the restrictions imposed by the Government of China to curb the spread of the virus, this study assessed that socially distanced employees might show weaker psychological and mental health. The fact that employees, like other humans, want a basic need for connectedness. Due to imposed lockdown and forced stay at homes during the Pandemic, less connectedness may negatively impact their health. This study's outcomes are consistent with the prior study, such as less social connectedness and socialization have been associated with employees' better mental health and performance (33).

Social connectedness is a basic human need. Previously, it was also noted that the social connectivity of employees has a positive effect on motivation and health of employees, and the employees who were devastated due to disruptive social ties with their friends and family, were at the stake of bad and deteriorated health. These outcomes on health showed serious impacts on their job performance (34). This study's results of testing fear of COVID-19 revealed that such fears had a significant association with employees' health. These consequences were negatively associated with employees' health. The results proved that fears contribute to stress on employees' mental and overall health. The employees who fear testing for COVID-19 may downgrade their health.

The symptoms of depression in employees arise due to the fear of testing COVID-19 positive. Some researchers in the recent past have also looked into such relationships where an increased level of COVID-19 fear negatively impacted the overall health of employees (41). Similarly, a recent study explored the relationship of fear of COVID-19 with psychological symptoms of depression and elevated levels of anxiety among employees. The study revealed that fear of COVID-19 was strongly associated with disturbed health of employees of healthcare in China (42). This study also looked into the relationship between employees' health and task performance. The results revealed that employees' health is linked with their task performance.

The results indicated that employees' well-being and health are strongly associated with their productivity and performance at the organizational level. Unhealthy employees are prone to several health disorders which restrict them from functioning properly and delivering performance at the organizational level. The fact is that employees' task performance is regarded as employees' efforts toward accomplishing the given task efficiently. Due to compromised health, employees are unable to deliver the assigned tasks efficiently. Some of the researchers also obtained similar results indicating that employees' task performance is an outcome of their general health. Due to the depression and anxiety developed during COVID-19, employees fell short of maintaining their health which ultimately affected their task performance (48). Their deteriorated task performance may also be disturbed due to other factors like working from home and social isolation at work.

The current research also examined the mediating effects of psychological strains between less social connectedness, fear of testing COVID-19 positive, and employees' health. The results revealed that psychological strains strongly and negatively mediated the relationships between less social connectedness, fear of testing COVID-19 positive, and employees' health. It was first identified that less social connectedness and fear of testing COVID-19 positive was significantly but negatively associated with employees' health. The role of psychological strain added to these relationships. Psychological strain is evaluated in terms of depression, anxiety, and stress, and all of these contribute unanimously to the psychological strain of employees at work (23, 42). Previous studies also evaluated the mediating role of psychological strain regarding the outcomes of quality of life of employees and got significant results (24).

### Theoretical and practical implications

The study's first and foremost theoretical contribution is examining the mediating role of psychological strain between less social connectedness of employees, fear of testing COVID-19 positive, and employees' health. Furthermore, the present study offers a comprehensive model for measuring a thorough relationship between less social connectedness and employees' health working in the electronics industries of China. The present study proposed that the lesser the social connectedness of employees at work, the worse will be the employees' health, which significantly contributes to their task performance. The current study's findings confirmed this notion and extended the literature on the lesser social connectedness construct and its negative consequences on employee health.

If employees are given more chances for social connectivity, it may improve their health and ultimately improve their task performance. Some of the practical implications of the study are as follows. Firstly, the organization's management working in electronic industries must show concern toward the overall health of employees by offering them regular opportunities to connect with their colleagues socially. The management should also focus on providing sessions to their employees about eliminating the fear of testing COVID-19 positive from their brains. This step would enhance the overall psychological health of the employees toward their task performance.

Secondly, China's electronics industry should promote a culture of providing a favorable environment that flourishes employees' performance toward the assigned tasks. Organizations can avoid psychological strain by facilitating the employees in terms of their meet-ups with their colleagues to enhance their working ability to achieve performance at the organizational level. This act would lead to higher performance and satisfaction among the employees. Thirdly, the organizations have to be careful about depression, anxiety, and stress symptoms which are found to weaken employees' health and ultimately deteriorate their performance.

### Limitations and future directions

One of the limitations of the study is related to the target population. This study only included the employees of the electronic industry of China; therefore, the future study can conduct on employees of multinational firms. Another limitation of the study is the small sample size which might affect the generalizability of the study. Hence, future research can enlarge the sample size or may conduct a longitudinal study to validate the present study outcomes. Moreover, the study was conducted in China, which could be the limitation of the present study; thus, the future study can include other regions or other Asian countries to examine the present study model. The current study predicted task performance as a whole construct. Therefore, to understand the model in depth, future studies can examine other performance attributes like the contextual performance of employees and job satisfaction, and turnover intention can also be a part of future research. Furthermore, the researchers can work on the advanced technologies related to the COVID-19 as Fuzzy Inference System and Machine Learning techniques, Blockchain-based digital twins in future.

## Conclusion

COVID-19 has affected the World population badly. The private business and public sectors are devastated by its detrimental effects globally. The organizations had to face many challenges to cope with the effects of COVID-19 in recent times. In this regard, almost all organizations considered various strategies to curb the speed of devastation due to COVID-19. These measures are equally taken at governmental and individual levels. Most of the efforts focused on the social distancing approaches to fighting the spread of this virus. These measures are taken to break the chains of spread. Some countries are still following the zero spread policies, and China is the most prominent participant in pursuing this strategy. Some countries follow a mixed approach to testing and maintaining the corporate sector for functioning. The corporate sectors are directly related to the economy of a country.

However, the lockdowns and social isolation approach badly affect the productivity progress in these corporations. COVID-19 has produced several stressors that affected their work performance in these organizations. This study tried to determine the impact of some of these stressors on employees' task performance. This study revealed that less social connectedness of employees due to these policies has a negative association with employees' health. Similarly, fear of testing COVID-19 positive had a significant and negative association with employees' health. This study also concluded that employees' health is directly related to their task performance. Moreover, the psychological strain adds more to it to worsen employees' health, restricting them from performing efficiently at the workplace because it mediates the negative relationship between less social connectedness and employee health and testing fear and employee health. Therefore, the current study contributes to the literature about devising policies which may target the employees' health and restrict the stressors which contribute negatively to employees' health.

## Data availability statement

The original contributions presented in the study are included in the article/supplementary material, further inquiries can be directed to the corresponding author.

## Ethics statement

The studies involving human participants were reviewed and approved by Shandong University of Arts, China. The patients/participants provided their written informed consent to participate in this study. The study was conducted in accordance with the Declaration of Helsinki.

## Author contributions

YZ: conceptualization, data collection, and writing the draft. The author agreed to the submitted version of manuscript.

## Conflict of interest

The author declares that the research was conducted in the absence of any commercial or financial relationships that could be construed as a potential conflict of interest.

## Publisher's note

All claims expressed in this article are solely those of the authors and do not necessarily represent those of their affiliated organizations, or those of the publisher, the editors and the reviewers. Any product that may be evaluated in this article, or claim that may be made by its manufacturer, is not guaranteed or endorsed by the publisher.

## Appendix 1

**Table A1:** Correlations among model variables.

**Construct**	**1**	**2**	**3**	**4**	**5**
Less social connectedness					
Testing fear	0.496^**^				
Psychological strain	0.737^**^	0.499^**^			
Employee health	–0.602^**^	–0.517^**^	–0.703^**^		
Task performance	0.592^**^	–0.384^**^	0.717^**^	0.543^**^	

**p < 0.01.
